# A comprehensive framework for solution space exploration in community detection

**DOI:** 10.1038/s41598-025-22046-7

**Published:** 2025-10-31

**Authors:** Fabio Morea, Domenico De Stefano

**Affiliations:** 1 Area Science Park, Padriciano 99, Trieste, Italy; 2 University of Trieste, Piazzale Europa 1, Trieste, Italy

**Keywords:** Network analysis, Community detection, Solution space, Outliers, Horizon project networks, Statistics, Computational science

## Abstract

Community detection algorithms are essential tools for understanding complex networks, yet their results often vary between runs and are affected by node input order and the presence of outliers, undermining reproducibility and interpretation. This paper addresses these issues by introducing a framework for systematic exploration of the solution space, obtained through repeated runs of a given algorithm with permuted node orders. A Bayesian model assesses convergence, estimates solution probabilities, and provides a defensible stopping rule that balances accuracy and computational cost. Building on this process, we propose a taxonomy of solution spaces that offers clear diagnostics of partition reliability across algorithms and a shared vocabulary for interpretation. Applied to a real-world network, the approach shows that different algorithms produce various types of solution space, highlighting the importance of systematic exploration of the solutions before drawing scientific conclusions.

## Introduction

Networks are widely used for representing complex systems where a set of nodes are connected by edges and are ubiquitous across diverse scientific fields^1^, including social sciences^2^, biology^3^, science of science^4,5^, neuroscience^6,7^, and innovation studies^8,9^.

Networks often exhibit the property of having a community structure, that is, their nodes are organised into groups, called communities, clusters, or modules^10^. Therefore, nodes can be partitioned into either disjoint or overlapping sets of vertices such that the number of edges within a set exceeds the number of edges between any two sets by some reasonable amount^11^. Discovering such tightly knit groups of nodes is crucial in many applications^12^. For example, in innovation diffusion networks, identifying significant and stable communities helps reveal knowledge flows between organisations or regions, shedding light on the underlying aggregation mechanism at play^13^. Community detection methods are often used for such task, and the key requirements are that the results must be independent of contingent factors (such as software implementation or ordering of the input data) and tested for validity.

Community detection typically assumes that a network can be partitioned into distinct communities and that a single optimal solution can be identified. It is clear that different algorithms often produce varying results due to methodological differences. However, under specific conditions, an algorithm may yield diverse outcomes depending on their inherent stochastic approach.

The present paper explores the importance of examining the solution space in the most commonly used community detection algorithms, highlighting its role in achieving reliable results when dealing with real-world networks. In this setting there are three issues that are still not extensively explored in the complex networks literature, namely: multiplicity of solutions, outliers handling, and input ordering bias.

In networks with fuzzy or complex community structures, significant variability in results can occur both across different algorithms and within repeated runs of the same algorithm.

Outliers, nodes that do not clearly belong to any community, are a critical yet under-explored aspect of community detection. Many algorithms either disregard outliers or assign them to existing communities, potentially distorting the detected structure. Proper identification and treatment of outliers are essential for generating accurate partitions and understanding their role in network dynamics.

Input ordering bias, where the sequence of nodes and edges affects algorithmic output, poses a significant challenge in community detection. Ideally, results should depend solely on the network’s intrinsic structure, not on an arbitrary processing order. However, several popular community detection algorithms are sensitive to this bias, leading to inconsistencies and affecting the reliability of the results. Mitigating this issue is essential to ensure that detected communities accurately reflect the network’s inherent topology rather than artifacts of computation.

To address these challenges, this study proposes a comprehensive methodology for exploring the solution space of community detection algorithms, coupled with a taxonomy to classify different types of solution spaces. This paradigm allows for a deeper understanding of the variability, biases, and anomalies present in network partitions, paving the way for more robust and meaningful analyses.

The remainder of this paper is structured as follows: Section 2 discusses related works addressing the quality issues in community detection results; Section 3 presents the methodological framework for solution space exploration, including the proposed taxonomy and outlier detection approach; Section 4 illustrates the methodology through synthetic and real-world network experiments; Section 5 concludes with a discussion of findings and future research directions.

## Related works

Several methods have been proposed to detect meaningful community structures based on the density of their internal connections ^14–16^. This principle is quite general although other attachment and aggregation mechanisms are possible in social networks ^17^. The main strategies for identifying an optimal partition include the detection of actors or edges with high centrality ^18^, optimisation-based algorithms ^19^, genetic algorithms^20,21^, statistical inference using stochastic block models ^22^. Furthermore, a new class of community detection methods has emerged that exploits node semantics or node attributes in addition to network topology. According to the taxonomy proposed by ^23^, these include graphical model-based community detection, deep learning-based community detection, as well as node embeddings ^24^.

Although many of these methods focus on partitioning networks into non-overlapping communities, there is a diverse range of variants, including hierarchical clustering^25,26^, which captures structures at different scales, overlapping communities^27–29^ and mixed-membership communities^30^, where a node can belong to more than one community, as well as a combination of overlapping and non-overlapping communities^31^.

However, probably due to their ability to produce easily interpretable results, optimisation methods and modularity based approaches that generate non-overlapping partitions are still widely used.

Despite their importance in practical settings, few studies focused on the issues discussed in the present paper or on the exploration of the solution space determined by the stochastic nature of such approaches.

Solution quality issues have been discussed in terms of the degeneracy of modularity maximisation algorithms^32^ and resolution limit^33^. Other papers used topological information to measure the quality of community structure identified in large scale networks^34^. More recent works have also delved into the multiplicity of solutions arising from community detection methods, mapping out how solutions can vary across different algorithm runs and parameter settings^35,36^. A number of contributions, focused on exploiting such characteristic, proposed the use of consensus clustering based approaches^37,38^.

An additional focus is to examine the behaviour of community detection algorithms when confronted with outlier nodes, which are nodes that display significantly different behaviour compared to the rest of the network^39^. Previous studies have focused on anomaly and outlier detection^40^, especially in the context of online social networks^41,42^. In our framework, we explicitly define outliers as nodes whose community membership changes frequently across repeated runs of community detection algorithms. However, the way such cases are handled in many widely used community detection algorithms remains under explored.

Input ordering bias has been discussed in the literature, notably by^32,43,44^ focusing mainly on modularity-based methods.

In this paper, we aim to generalise the results on the above discussed issues to any algorithm, and to devise a procedure that mitigates particularly the input-ordering bias, while improving the stability and reliability of results.

## Methodology

Let $G=(V,E)$ be a graph with $n_v = |V|$ vertices and $n_e = |E|$ edges (for a list of key symbols and notations used throughout the manuscript, see the Supplementary Table S1 ). A community *C* is a subnetwork of *G* where nodes are more densely connected to each other than to the rest of *G*. A partition *P* is a set of *k* disjoint subnetworks $C_1, \ldots , C_k$ whose union is equal to *G*. A community detection algorithm $\mathcal {A}(G, \rho ) \rightarrow P$ is a function that takes as input a graph *G* and one or more parameters $\rho$, and returns a partition *P*. Ideally, each community detection algorithm discussed in Section 2 should yield a single valid partition when applied with a given set of parameters. However, in practice, for large and dense networks, $\mathcal {A}$ may produce different partitions, $P_i \ne P_j$ in repeated runs.

### Solution space definition and characterisation

The solution space $\mathbb {S} = \{P_1, P_2, \ldots , P_{n_s} \}$ is the set of all *n*_*s*_ unique partitions that $\mathcal {A}$ produces in *t* trials or runs.

**Fig. 1:**
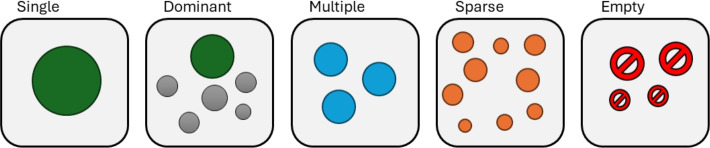
Taxonomy of solution spaces generated by community detection algorithms. The framework classifies partitions into five categories based on posterior probabilities and convergence behaviour: Single (only one stable partition), Dominant (one prevailing solution with probabilistic separation from others), Multiple (several comparable solutions with no clear separation), Sparse (many low-probability partitions, often incoherent), and Empty (degenerate or non-valid partitions).

Our research introduces the notion that $\mathbb {S}$ can be characterised through an iterative exploration process. In this process, $\mathcal {A}$ is applied repeatedly under random permutations of the graph’s input ordering—permutations that preserve the topology and information content—and the resulting partitions are modelled probabilistically. We also propose a taxonomy to classify $\mathbb {S}$ into different categories (depicted in Fig. 1), based on the number of unique solutions, their probabilities and the convergence of the process.

The exploration process, detailed in Algorithm 1, consists of repeated trials guided by the convergence of a Bayesian model $\mathbb {M}$, based on the Dirichlet-Multinomial distribution.

The model maintains both counts $\textbf{c} = (c_1,\ldots ,c_k)$ tracking how often each solution $P_i$ is observed and their corresponding probability estimates $\hat{\textbf{p}} = (\hat{p}_1,\ldots ,\hat{p}_k)$, that quantify the likelihood of each partition. The marginal Beta distributions provide credible intervals $[\hat{p}_i^\ell ,\hat{p}_i^u]$ that quantify the uncertainty in each probability estimate.

The discovery process consists of repeated trials, whose independence is ensured through algorithmic stochasticity and permutation of network inputs (Algorithm 1, step 4). However, model $\mathbb {M}$ relies on the exchangeability of observations, a condition that is justified by the same mechanisms, allowing effective sampling from the posterior distribution.

At the beginning, the model is initialised with a single dimension and a weakly informative prior, to reflect the belief that at least one solution exists. At each trial, the algorithm returns a partition $P_t$ that is compared to all previously observed solutions using Normalised Mutual Information (*NMI*), a measure of partition similarity that is invariant to community label permutations. If $P_t$ matches an existing solution ($\textrm{NMI}(P_t, P_j) = 1$ for some *j*), the corresponding count is incremented. When $P_t$ is novel ($\textrm{NMI}(P_t, P_j) < 1$ for all existing $P_j$), the model is expanded to track this new solution.

After $t$ trials, the posterior distribution over solution probabilities is given by:

$$\textbf{p} \mid \textbf{c} \sim \text {Dirichlet}(\gamma _0 + c_1, \ldots , \gamma _0 + c_k)$$

where $c_i$ is the number of times solution $P_i$ has been observed, and $\gamma _0$ is the prior count assigned equally to all solutions.

The exploration process is terminated either at convergence or upon reaching the maximum number of trials $t_{\max }$.

Convergence occurs when the posterior renders the solution space statistically unambiguous under one of two conditions: (i) *Stabilisation*, when $\max _i (p_i^u - p_i^\ell ) \le \delta$, where $\delta >0$ is a tolerance threshold controlling the required precision of probability estimates; or (ii) *Separation*, when $\exists \, i^\star :\; p_{i^\star }^\ell > \max _{j\ne i^\star } p_j^u$, indicating that one solution is unambiguously dominant.

If neither condition is met within $t_{\max }$ trials, the process stops in an intermediate regime where new solutions may continue to emerge sporadically. The thresholds $\delta$ and $t_{\max }$ balance computational cost against the benefit of further exploration. In this study, we set $\delta = 0.1$ and $t_{\max } = 50$, a choice supported by empirical evidence, as most cases converge earlier and non-convergent ones are *Sparse* (see Fig. 1 and Section Taxonomy for the definition) where additional trials would not alter the solution space classification.

**Algorithm 1:**
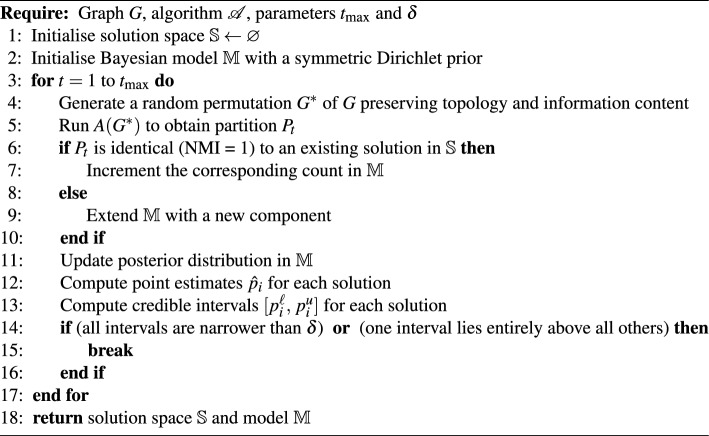
Solution space exploration.

### Taxonomy

The taxonomy proposed in Fig. 1 can be formally defined as follows:

- **Single** The exploration process achieves stabilisation with only one valid partition ($n_s = 1$). In this case, the process terminates at convergence, meeting the stabilisation criterion.
- **Dominant** Multiple valid partitions exist ($n_s > 1$), but one exhibits a decisive separation: $i^* = \arg \max _i \hat{p}_i$ where $p_{i^*}^l > \max _{j \ne i^*} p_j^u$. The process terminates at convergence, satisfying the separation criterion.
- **Multiple** Several partitions are observed ($n_s > 1$) with comparable probabilities (credible intervals are overlapping). No solution achieves a decisive separation and $\max _i(p_i^l) < 0.5$.
- **Sparse** A large number of solutions is observed ($n_s \approx t$), each with low individual probability ($p_i \approx 1/n_s$). Typically, the exploration process terminates at $t_{max}$ without achieving convergence.
- **Empty** No meaningful partition is observed, either because the algorithm produces no valid solutions ($n_s = 0$) or because all solutions violate basic community requirements, such as all nodes grouped in one community ($k = 1$), all nodes as singletons ($k = n_v$), or communities are internally disconnected.

The solution space taxonomy serves as a decision aid for downstream analysis, indicating if a single, representative partition can be credibly reported, and, conversely, if the evidence supports the absence of credible community structure.

In the Single case, the analysis is straightforward, and the unique partition can be regarded as representative. The Dominant case is more nuanced: a prevailing solution coexists with credible alternatives. Whether the most probable partition can be treated as representative is context-dependent and should be justified, for instance, by clear probabilistic separation from the alternatives. When the solution space is classified as Multiple, it is important to check for symmetry: if *G* admits *k* symmetry equivalent partitions, no single solution is expected to exceed $\approx 1/k$ posterior probability. When a single representative partition must be reported in Dominant or Multiple solution spaces, a consensus procedure can be used to synthesise information across the competing solutions. A Sparse solution space can arise in two ways. (i) Solutions may be largely similar, differing only in minor details; they then form one or a few coherent clusters (with possible outliers), indicating an underlying structure that can be summarised via consensus. (ii) Partitions are mutually dissimilar, which may signal a lack of a credible community structure. This situation is easily observed when applying community detection algorithms to random graphs: each run returns a partition, yet the set of partitions is collectively incoherent.

In the following we discuss other possible issues affecting the solution space, that are input ordering bias and the identification of outlier nodes in the observed network.

### Input ordering bias

Although graphs are mathematically unordered objects, software implementations necessarily process ordered data structures such as nodes and edge lists, or adjacency matrices. As a result, many community detection algorithms become sensitive to otherwise irrelevant features such as node or edge indexing, order of iteration, or tie-breaking. We refer to this phenomenon as *input-ordering bias*.

Input-ordering bias can be detected by comparing solutions generated by $\mathcal {A}(G)$ with those generated by $\mathcal {A}(G^{*})$ where $G^{*}$ is an information preserving permutation of *G* obtained only by reordering nodes and edges. If $\mathcal {A}$ is order invariant, the outcomes should be statistically indistinguishable.

If input ordering bias is not addressed, runs performed on a fixed node order may undersample the solution space: fewer distinct partitions are discovered, and the apparent dominance of some solutions may be overstated. Our exploration procedure implements systematic, information preserving permutations at every trial (Algorithm 1, Step 4). As already mentioned, this double randomisation—algorithmic stochasticity plus independent permutation—mitigates order dependent artifacts, expands coverage of the solution space, and justifies the exchangeability assumption underlying our statistical model.

### Outliers

Our approach to outlier detection is coherently grounded in the data and framework generated through solution space exploration, providing a straightforward method for identifying node outliers. The first step is to compute a *Pairwise Agreement Matrix* $\Gamma$, an $n_v \times n_v$ symmetric matrix with entries:

$$\gamma _{uv} = \sum _{i=1}^{n_s} \hat{p}_i \cdot \chi _i(u,v), \quad \Gamma = [\gamma _{uv}]$$

where $\chi _i(u,v)=1$ if nodes *u* and *v* belong to the same community in partition $P_i$, and 0 otherwise; $n_s$ denotes the number of unique partitions in the solution space $\mathbb {S}$, and $\hat{p}_i$ is the estimated probability of partition $P_i$.

To assess whether a node *v* is an outlier, we examine its *pairwise agreement profile*, defined as the *v*-th row of $\Gamma$, which is a vector of length $n_v$. Its entries $\gamma _{uv}$ quantify the frequency-weighted co-occurrence of *v* with each other node in the network. Entries equal to zero indicate pairs that never co-occur in the same community and can be excluded from the calculation.

The analysis of $\gamma _{uv}$ offers a straightforward means of quantifying outlier tendencies without extensive algorithmic sophistication, making it particularly valuable for exploratory analysis and initial outlier screening. For example, $\overline{\gamma }_v^{(+)}$ as mean of the non-zero entries of $\gamma _{uv}$ is a single number associated to each node. Stable (i.e., non-outlier) nodes have $\overline{\gamma }_v^{(+)}$ values close to 1.0 and consistently appear with the same neighbours, while low values of $\overline{\gamma }_v^{(+)}$, indicates nodes that are assigned to different communities within the solution space.

## Results and discussion

### Experimental design and test networks

**Fig. 2:**
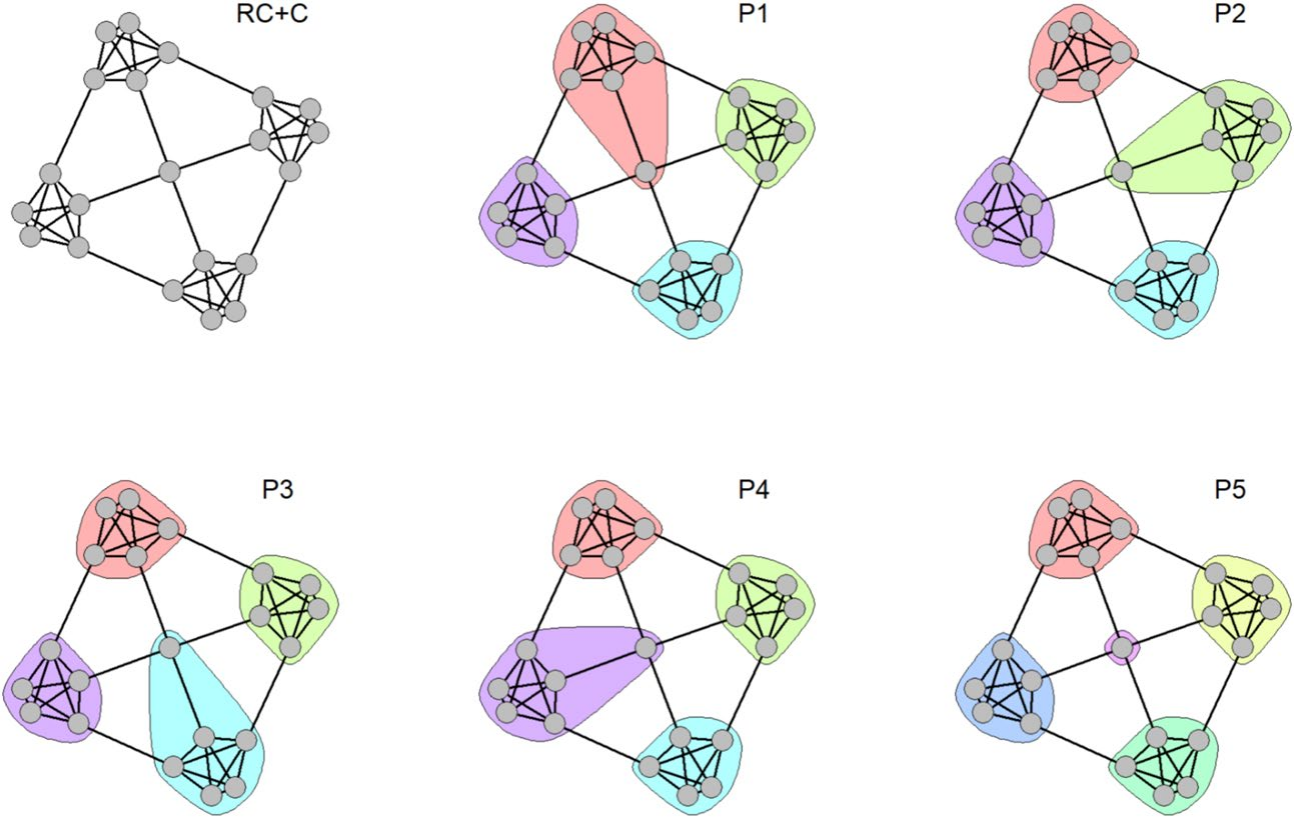
Ring of cliques with central outlier (${RC{+}C}$) and resulting partitions. The ${RC{+}C}$ network comprises four cliques and a central outlier node connected to all cliques. Partitions $P_1$ through $P_4$ assign the outlier to one clique, introducing imbalance. Partition $P_5$ isolates the outlier as a single-node community, a configuration not all algorithms can produce.

To test the proposed methodology, a specific test network is used: a Ring of Cliques with a central outlier (${RC{+}C}$), shown in Fig. 2. A Ring of Cliques (RC) is a network composed of $n_c$ cliques of size $c_s$, i.e., fully connected groups of nodes that naturally form distinct communities, arranged in a circular pattern. Each clique is isolated in terms of connectivity, making the community structure clear and straightforward. The ${RC{+}C}$ builds on the basic RC structure by introducing an outlier: a “central” node, connected to each clique.

Although the community structure is visually obvious to human observers, algorithms operate without prior knowledge of the network structure. As a result, they may produce partitions that overlook the symmetry of the central node. Additionally, some algorithms can produce different solutions with each independent run and may exhibit an input ordering bias.

Most algorithms are likely to generate partitions such as $P_1$, $P_2$, $P_3$, or $P_4$ shown in Fig. 2, where the outlier is incorporated into one of the cliques. However, these solutions are inherently flawed, as they unfairly favour one clique over the others. Alternatively, a solution like $P_5$, where the outlier forms a separate community, may appear more equitable. Nevertheless, not all algorithms are capable of producing such a partition, as it requires accepting the assumption that a single node can constitute a valid community.

The tests were carried out using six algorithms: Edge Betweenness (EB), Louvain (LV), Leiden (LD), Label Propagation (LP), Infomap (IM) and Walktrap (WT), selected for their widespread use in applications, ready availability in the iGraph^45^ library for the R programming language^46^, and their representation of different approaches to community detection.

In the following, we briefly describe the main characteristics of such algorithms.

- **EB** The Edge Betweenness (EB)^18^ algorithm assumes that edges bridging different communities exhibit high betweenness centrality, defined as the frequency with which an edge lies on shortest paths between pairs of nodes. At each step, the edge with the highest score is removed values are recalculated, and the process yields a hierarchical dendrogram of partitions. The final partition is typically chosen according to an external optimality criterion, though no unique rule is prescribed. EB is computationally demanding and does not scale to large networks.
- **LV** The Louvain (LV) algorithm^47^ is an agglomerative method that optimises modularity using a greedy approach. Initially, each node is assigned to a separate community; nodes are then iteratively moved to the community of one of their neighbours, maximising modularity, until no further improvement can be made. A resolution parameter (*r*) controls the granularity of partitions: $r>1$ produces smaller and more numerous communities, while $r<1$ produces larger and fewer ones. LV is fast and scales efficiently to large graphs, but it has the drawback that it may produce communities that are internally disconnected. The algorithm is stochastic, as nodes are examined in random order, and it converges only to local rather than global maxima of modularity; consequently, each run can yield a different partition.
- **LD** The Leiden (LD) algorithm^48^ is an improvement of LV that addresses internally disconnected communities. After the initial greedy optimisation phase, LD adds a refinement step that splits any disconnected communities, ensuring all detected communities remain internally connected. Like LV, LD is stochastic due to random node examination, and can yield different partitions across runs.
- **IM** The Infomap algorithm^49,50^ detects communities by minimising the description length of a random walker’s trajectory under the map equation. The optimisation employs stochastic search heuristics, so results vary across runs.
- **WT** Walktrap^51^ is a hierarchical clustering algorithm based on the assumption that nodes within a community are likely to be connected by shorter random walks. The algorithm begins with each node in its own community and iteratively merges pairs of communities based on a distance measure derived from random walks, producing a hierarchical dendrogram of partitions. A user-defined parameter *s* defines the length of the random walk to be performed, controlling the resulting community size.
- **LP** Label Propagation (LP) relies on the notion of proximity or neighbourhood relationships, as discussed in^52^. Initially, each node is assigned a unique community label, then the nodes are iterated through in a random sequential order, and each node adopts the label that is most prevalent among its neighbours. This process continues until each node shares the label of the majority of its neighbours. The algorithm is stochastic, as both the sequence of node updates and the resolution of label ties are random, so different runs often yield different partitions.

### Results on artificial networks (Ring of Cliques)

**Fig. 3:**
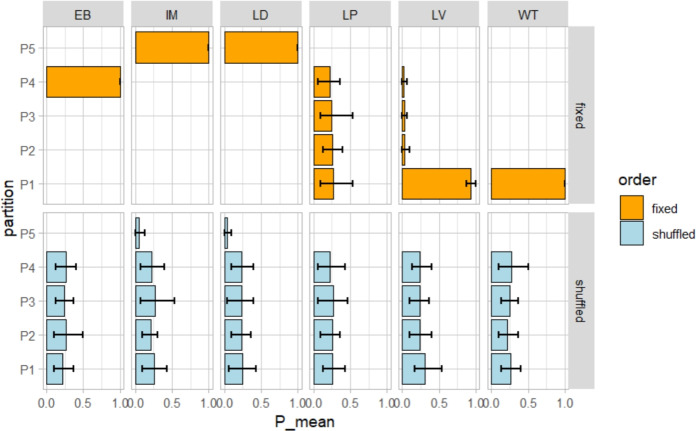
An illustration of multiplicity of solutions and input-ordering bias. Test carried out on a Ring of Cliques with *nc* = 4, *cs* = 5 with a central node. The bars show the frequency (out of 1000 runs) of assigning the central node to each of the 5 possible partitions (P1-P5). The network structure creates 5 equivalent solutions that are equally likely, so frequencies should be uniformly distributed if algorithms are unbiased. Orange bars: fixed node ordering. Blue bars: permuted node ordering at each iteration.

We analyse the behaviour of the selected algorithms regarding their tendency to generate multiple solutions, their strategies for managing outliers, and their susceptibility to input ordering bias. The results are presented in Fig. 3 and summarised in Table 1.

We observe that most algorithms exhibit a noticeable input-ordering bias, with the exception of LP. Specifically, when applied to ${RC{+}C}$, IM and LD assign the centre to a single-node community ($P_5$). EB and WT always aggregate the center to one of the communities ($P_4$ and $P_1$, respectively), while LV strongly favour the community $P_1$. However, when applied to a randomly permuted ${RC{+}C}^{*}$, each algorithm produces a less biased result.

**Table 1:** Comparison of community detection algorithms across the identified issues. Three key behavioral characteristics are reported: (1) Multiple solutions—whether the algorithm produces different solutions at each run regardless of input order, (2) capability to assign a single node to its own community, and (3) sensitivity to input node ordering.  EB = Edge Betweenness, LV = Louvain, LD = Leiden, LP = Label Propagation, IM = Infomap, WT = Walktrap.

**Feature/Method**	**EB**	**LV**	**LD**	**LP**	**IM**	**WT**
(1) Multiple solutions	No	Yes	No	Yes	No	No
(2) Single-node community	No	No	Yes	No	Yes	No
(3) Input ordering bias	Yes	Yes	Yes	No	Yes	Yes

As an illustrative example of outliers detection, in the ${RC{+}C}$ network of Fig. 2, all nodes within the cliques always classified as stable communities with $\overline{\gamma }_v^{(+)} = 1.0$ relative to each other, whereas the central outlier node has $\overline{\gamma }_v^{(+)} = 0.25$ with any clique node, meaning it determines the actual differences between the solutions.

**Fig. 4:**
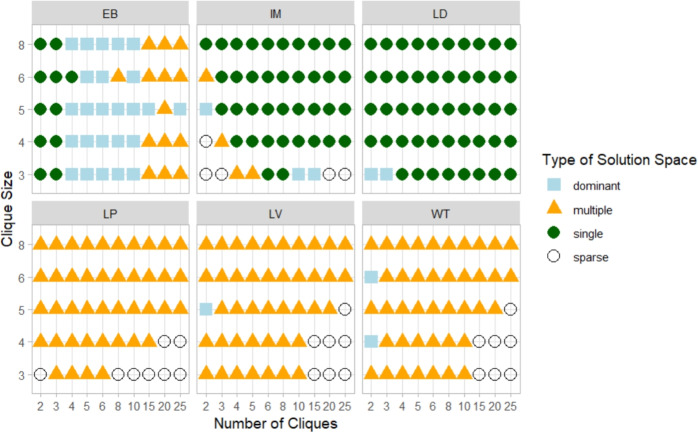
Prevalent type of solution space generated by different algorithms.

The solution space exploration methodology was applied to a set of ${RC{+}C}$ networks, with the number of cliques *nc* varying between 2 and 25 and the clique size *cs* ranging from 3 to 8. This setup tests the ability of algorithms to generate different types of solution spaces. The results are presented in Fig. 4 and summarised in Table 2.

The results indicate that algorithms exhibit a prevalent tendency to generate specific types of solution spaces, but they also reveal inherent limitations. For instance, most diagrams display a Sparse solution space, particularly in cases with 2 or 3 large cliques, as seen in the bottom right corner of Fig. 4. Additionally, when $nc = 2$, an extreme case of a graph composed of two cliques connected by one edge, particularly for smaller clique sizes, most algorithms struggle to detect a single solution, with performance improving as clique size increases, highlighting their limitations in handling minimal community structures.

Beyond the Single case, comparing partitions is informative, as it reveals whether multiple solutions differ only marginally or diverge substantially, potentially forming clusters of alternative solutions. Such comparisons can be conducted using similarity measures between partitions, for example the Normalised Mutual Information (*NMI*), combined with the estimated probabilities of partitions in the solution space. For example, in the ${RC{+}C}$ network (Fig. 2), the four partitions $P_{1}$–$P_{4}$ constitute a Multiple solution space, occurring with comparable estimated probabilities ($\approx 0.25$ each) and differing only in the assignment of the central node.

**Table 2:** Comparison of methods across the identified issues.

**Algorithm**	**EB**	**LV**	**LD**	**LP**	**IM**	**WT**
Prevalent type	Dominant	Single	Single	Multiple	Multiple	Multiple
Single $(cs = 5)$	$nc<4$	$nc>3$	Always	Never	Never	Never
Sparse	Never	$cs<5$	Never	$cs<6$	$cs<5$	$cs<6$

### Application on a real network

To illustrate how the exploration of the solution space on real data allows replicable and interpretable results, we present an example of application to a large, dense network from the Horizon Projects Network dataset. The source of data is CORDIS (Community Research and Development Information Service), the European Commission’s primary public repository and portal to disseminate information on all EU-funded research projects and their outcomes (https://cordis.europa.eu/). This dataset captures collaboration linkages between organisations participating in EU-funded research projects between 2015 and 2027 in various domains; a comprehensive description of the dataset is provided in^13^. For illustrative purposes, we focus on the collaboration network emerging from projects in the hydrogen energy domain that start in 2024 and retrieved using hydrogen as keyword. We denote this network as $G_{2024}$. It is composed of 648 nodes (nodes represent organisations, in their role of partners of one or more Horizon projects on hydrogen) and over 6157 edges denoting collaborative relationships—i.e., two organisations participating in the same project within a given year. Edge weights reflect the funded amount granted to each collaborative project.

An overly simple approach to the analysis of $G_{2024}$ would be to apply a community detection algorithm (e.g., Louvain) and directly interpret the resulting partition. However, as discussed above, we argue that each run of an algorithm should be regarded as generating a sample from the solution space; therefore, we applied all the algorithms mentioned above, and the results are summarised in Table 3.

**Table 3:** Summary of solution space exploration on the Horizon network $G_{2024}$. The six algorithms yield markedly different outcomes: Walktrap (WT) converges to a Single solution, Label Propagation (LP) and Louvain (LV) generate Sparse solution spaces, producing a different solution at each run. Leiden (LD) results in an Multiple solution space, where all solutions are degenerate. Infomap (IM) produces a Multiple solution space with two solutions of overlapping probabilities clearly separated from the others. Finally, Edge Betweenness (EB) could not deliver any solution due to excessive computational cost. Parameters: $\delta = 0.1$ and $t_{max} = 50.$

	**WT**	**LV**	**LD**	**LP**	**IM**
Convergence	Stabilisation	Stabilisation	max. trials	stabilisation	max. trials
Trials	18	29	50	29	50
Total solutions	1	29	11	29	13
Valid solutions	1	24	0	20	13
Type of solution space	Single	Sparse	Multiple	Sparse	Multiple

This network represents a challenging case, and each method yields a distinct type of solution space. Walktrap (WT) identifies a single solution, and after 18 trials matches the stability condition. Louvain (LV) and Label Propagation (LP) generate a Sparse solution space, with a different solution at each trial, which reaches the termination condition after 29 trials. Both produce some non-valid solutions (specifically, some communities are internally disconnected). Leiden (LD) results in a Multiple solution space, and the exploration stops after $t_{max}$ trials. However, in this case all the solutions are degenerate, as the number of inter-community edges is greater than the number of intra-community edges, contradicting the general definition of community. If this is deemed as a relevant issue for the analysis, the solution space may be considered Empty. Finally, Edge Betweenness (EB) could not be computed in a reasonable time, confirming its impracticality on networks of this size.

**Fig. 5:**
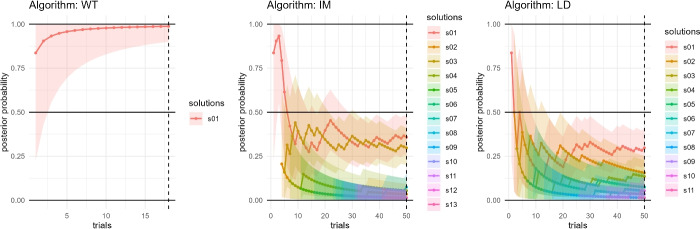
A graphical representation of the successive steps in the exploration of the solution space for the Horizon network $G_{2024}$ using three representative algorithms. Each curve shows the posterior probability $\hat{p}_i$ of solution $P_i$ as a function of the trial number *t*, with shaded areas denoting the credible intervals $[p_i^l,\,p_i^u]$.

**Fig. 6:**
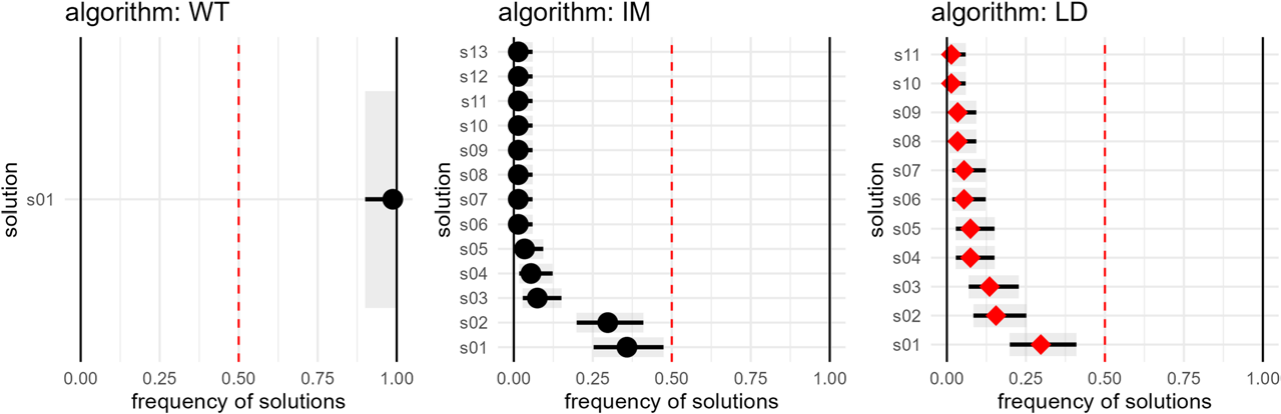
Distribution of solution frequencies and credible intervals for the Horizon network $G_{2024}$ obtained with three algorithms (Walktrap, Infomap and Leiden). Each point represents the estimated posterior probability $\hat{p}_i$ of solution $P_i$, with horizontal bars denoting the Bayesian credible intervals $[p_i^l,\,p_i^u]$ and the vertical dashed line marking the $\hat{p}_i = 0.5$ threshold.

Figure 5 presents the evolution of posterior probabilities $\hat{p}_i(t)$ together with credible intervals $[p_i^\ell ,p_i^u]$, for three representative algorithms (WT, IM and LD), revealing heterogeneous convergence behaviours. Walktrap (WT) converges rapidly by $t=18$, with shrinking intervals consistent with a Single solution space. Infomap (IM) oscillates among alternatives until two competing partitions of comparable probability emerge, exemplifying a Multiple case. Leiden (LD) remains unstable throughout and does not satisfy the convergence criteria, thus generates a Sparse solutions space; exploration halts at the maximum number of trials, $t_{\max }=50$. Figure 6 complements this view with the final distribution of solution probabilities and uncertainties: each point reports $\hat{p}_i$ with horizontal bars for $[p_i^\ell ,p_i^u]$, while the dashed vertical line at $\hat{p}_i=0.5$ serves only as a visual reference. These figures further illustrate why characterising the solution space is critical for downstream analyses that often rely on a single representative partition of *G*. In this example, a single run is defensible only for Walktrap (WT), which yields a single solution, whereas for Infomap (IM) and Leiden (LD) single-run outputs would overlook the multiplicity and instability revealed by the posterior and risk drawing misleading inferences. When the solution space is Dominant or Multiple and a single partition is nevertheless required, a consensus procedure is advisable, aggregating partitions and optionally weighting them by their posterior probabilities $\hat{p}_i$. In Sparse spaces, consensus may be appropriate only when solutions form one or a few coherent clusters; otherwise the evidence may support reporting the absence of a credible community structure, rather than forcing a single partition.

## Conclusions

This study underscores the necessity of exploring the full solution space in community detection, particularly when working with large and complex networks, and introduces a taxonomic framework to classify its structure based on the number of unique solutions, their relative frequencies, and convergence behaviour.

Exploration of solution space proceeds via repeated runs of a given community detection algorithm on the same network, with node order permuted at each run. A Bayesian model assesses convergence and estimates solution probabilities, yielding a defensible stopping rule that balances exploration accuracy and computational cost. The taxonomy classifies solution spaces into five types (Single, Dominant, Multiple, Sparse, and Empty) providing clear diagnostics of partition reliability across algorithms and a shared vocabulary for interpretation.

Our empirical analysis shows that most widely used algorithms are sensitive to input (node) ordering, challenging the assumption that outcomes reflect topology alone. Systematically permuting the input reduces this bias and yields more reliable partitions. More broadly, repeated exploration of the solution space reveals that a single run can miss important structure and even elevate a minority partition to apparent representativeness. The main limitation of the proposed framework is computational: the total runtime scales with the number of trials, making the method impractical at large scale.

Future work should assess how solution space exploration impacts applications across different domains and scales. This involves broadening empirical tests to include additional algorithms, examining the grey area between Multiple and Sparse solution spaces, and integrating quality and similarity metrics (e.g., modularity, NMI) to refine the treatment of cases that, in this paper, were more broadly defined as “degenerate” or “non-valid” solutions.

The methodology should also be tested across a wider range of domains, extending beyond the innovation network case presented here to fields such as biology, neuroscience, and other technological or organisational networks.

## Supplementary Information

Supplementary Information.

## Data Availability

All data and code used in this study are available under a CC-BY license. The sample network $G_{2024}$ is part of the Horizon Projects Network dataset (https://doi.org/10.5281/zenodo.13765372). The analysis was conducted using the R programming language, the package igraph (https://r.igraph.org/), and our package communities (10.5281/zenodo.13594209).
