# Supplementary material for: A comprehensive framework for solution space exploration in community detection
Source: Sci Rep. 2025 Oct 31;15:38148. doi: 10.1038/s41598-025-22046-7 (PMC12578937; doi:10.1038/s41598-025-22046-7)
Supplement: Supplementary file 1 — Supplementary Information. [file 41598_2025_22046_MOESM1_ESM.pdf]

Supplementary Information

This appendix provides a reference for the key symbols and notations used throughout the manuscript (Supplementary Table S1).

Symbol	Meaning
$G = (V, E)$	Graph (network) under study, with node set V and edge set E.
$n_v = \|V\|$, $n_e = \|E\|$	Number of nodes and E.
$C \subseteq V$	Community as a subset of V.
P	Partition of V into disjoint communities.
$\mathscr{A}(G, \rho)$	Community detection algorithm run on G with parameter(s) ρ.
$\mathbb{S}$	Solution space: set or all distinct partitions observed across trials.
t, $t_{\max}$	Trial index and maximum number of trials.
$\mathbb{M}$	Probabilistic model defined within a Bayesian framework, based on the Dirichlet-Multinomial distribution.
$\mathbf{c} = (c_1, \ldots, c_k)$	Counts: number of times each solution P_i is observed.
$\mathbf{p} = (p_1, \ldots, p_k)$	frequencies of solutions
$\hat{p}_i$, $[p_i^\ell, p_i^u]$	Posterior mean and credible interval for solution i.
δ	Convergence tolerance used in the stabilisation criterion.
$\mathit{\Gamma} = [\gamma_{uv}] \in [0, 1]$	Pairwise agreement matrix: fraction of trials assigning u and v to the same community.
$\gamma_v^{(+)}$	Node-level agreement score derived from $\mathit{\Gamma}$; used to flag candidate outliers.
$\chi_i(u, v)$	Characteristic (indicator) function: 1 if nodes u and v belong to the same community in partition P_i, and 0 otherwise.
$\mathrm{NMI}(P_i, P_j) \in [0, 1]$	Normalised Mutual Information between partitions P_i and P_j.
WT, LV, LP, LD, IM	Algorithm abbreviations: Walktrap, Louvain, Label Propagation, Leiden, Infomap.
RC	Ring of Cliques artificial network.
RC+C	Ring of Cliques artificial network with outlier node.
nc	Number of cliques in RC or RC+C
cs	size of cliques in RC or RC+C

Table S1. Symbols and notations used in the manuscript.
